# A Pooled Analysis of Growth and Tolerance of Infants Exclusively Fed Partially Hydrolyzed Whey or Intact Protein-Based Infant Formulas

**DOI:** 10.1155/2018/4969576

**Published:** 2018-11-01

**Authors:** Laura A. Czerkies, Brian D. Kineman, Sarah S. Cohen, Heidi Reichert, Ryan S. Carvalho

**Affiliations:** ^1^Nestlé Nutrition, Florham Park, NJ, USA; ^2^EpidStat Institute, Ann Arbor, MI, USA

## Abstract

**Background:**

For infants who are partially or exclusively fed infant formula, many options exist with compositional differences between formulas making choices difficult for caregivers and healthcare professionals. The protein in routine infant formulas differs by the source, fraction of cow's milk protein used, and degree of hydrolysis. All commercially available regulated infant formulas support growth and development, but different stool patterns have been observed based on formula composition. A pooled analysis of seven clinical trials was conducted to examine growth, stool consistency, and stool frequency of infants fed an intact cow's milk-based formula (CMF) or a partially hydrolyzed whey formula (PHF-W) from a single manufacturer.* Method*s. Individual subject data from seven infant formula growth studies (3 CMF, 4 PHF-W) were pooled and analyzed. All studies included healthy, full-term, formula-fed infants enrolled at 14 days of age with outcomes assessed over 4 months. Gains in weight and length to 4 months were analyzed using linear regression accounting for clustering within study. Outcomes of caregiver-reported stool consistency and frequency were analyzed using a longitudinal multinomial model.

**Results:**

Data from 511 infants were included (197 CMF, 314 PHF-W). There were no differences in weight gain between groups. There was no difference in length gain in girls fed PHF-W while boys fed PHF-W had a significant difference of +0.016 cm/month compared to boys fed CMF. Infants fed PHF-W had a significantly higher probability of soft and lower probability of hard stools as compared to infants fed CMF at each time point (p<0.001). Stool frequency was similar between groups.

**Conclusions:**

Infants fed CMF and PHF-W exhibit appropriate growth with comparable gains in weight and length through 4 months. More soft and fewer hard stools are observed in infants fed PHF-W compared to CMF. This difference could help to inform decision-making when choosing an infant formula.

## 1. Background

Breastfeeding is the ideal way to nourish an infant. However, for infants who are partially or exclusively fed infant formula, many options exist among formulas making choices difficult for both caregivers and healthcare professionals. The biggest compositional differences between formulas is the source of protein. All commercially available and regulated infant formulas provide adequate nutrition to support the critical growth of infants, but differences in protein source and degree of hydrolysis may lead to subtle but important differences in stooling patterns about which healthcare practitioners and caregivers should be aware. In the United States, routine infant formula can contain intact cow's milk protein or individual fractions of cow's milk protein that has been hydrolyzed (partially or completely broken down to smaller peptides). Partially hydrolyzed protein-based routine infant formulas are designed to be easy to digest and are frequently used in formula-fed infants with common feeding issues.

Partially hydrolyzed formulas contain cow's milk protein that has been processed through enzymatic and/or heat treatment to breakdown the protein present. Some manufacturers use a casein and whey combination while others use only whey. Presently the evidence suggests that partial hydrolysates tend to have beneficial effects on functional GI manifestations such as regurgitation and constipation, and these formulas may be considered as ‘solution' infant formulas when formula-fed infants experience tolerance-related issues [1].

Pediatricians' awareness of differentiating factors among these types of formulas for non-exclusively breastfed infants could be helpful for guiding parents in their decision-making around formula choice. Beyond general overall infant health, spit-up frequency, stool patterns, and colic are among the most important factors in parental consideration of infant formula choice [2, 3]. Infant stool consistency may also be an area of concern for parents, and thus understanding how different infant formula compositions affect stooling is important information to enable healthcare practitioners to assist their patients' caregivers with feeding decisions. The focus of this analysis was examining protein differences. Previous studies have shown that feeding infants with certain partially hydrolyzed formula leads to predominantly soft stools [4, 5]. To further explore this finding and better inform healthcare providers as well as caregivers, we conducted a pooled analysis of seven clinical trials to examine growth and tolerance as measured by stool consistency and frequency of infants fed either intact cow's milk formula (CMF) or a partially hydrolyzed whey-based formula (PHF-W) from a single manufacturer.

## 2. Methods

### 2.1. Data Collection

Seven studies were included in this pooled analysis. All studies were performed in accordance with the Declaration of Helsinki and were approved by an appropriate ethics committee prior to study enrollment. Informed consent was obtained from caregivers of all participants. All infants were exclusively formula-fed, full-term infants (≥37 weeks gestation) who were ≤ 14±3 days at the time of study enrollment. In all studies, subjects were required to have a birthweight between 2500-4500 g. Type of delivery (vaginal or Cesarean section) and history of breastfeeding was collected as part of the demographic information on subjects.

The purpose of this pooled analysis was to compare outcomes of infants fed PHF-W or CMF from a single manufacturer. In this manner, a pooled analysis was possible due to the availability of individual patient data. This is desirable in order to increase statistical power. All studies were selected after review of the archives of the manufacturer from data collected by studies of the manufacturer based on similarity in design and individual patient data availability, and consistency of data collection.

Patient compliance was available from all studies with an assignment in the original analysis of either meeting the intention to treat (ITT) or per protocol (PP) criteria. Data from subjects receiving CMF without probiotics and prebiotics were included for 3 studies conducted between 2004-2008 in Italy and France [6–8]. Data from subjects receiving PHF-W without probiotics and prebiotics were included for 3 studies conducted between 2000-2013 in the United States (US) [9–11]; one additional PHF-W study formula included a prebiotic [5]. All study formulas were manufactured by a single manufacturer (Nestlé Nutrition; Table 1). The PHF-W used in 3 studies was a formula comprised of 100% whey, partially hydrolyzed providing 0.67 kcal/ml and 2.2 g protein/100 kcal [5, 10, 11]. One PHF-W study included a formula with the same protein source and energy density, but with 2.39 g protein/100 kcal [9]. The CMF studies all included a protein source that was 70:30 whey:casein, 0.67 kcal/ml, and provided 1.8 g protein/100 kcal [6–8]. Formulas used in all studies consisted of protein, carbohydrate and fats with vitamins and minerals in amounts intended for full nutritional support of full-term infants. Fat content was similar between the two formulas consisting of vegetable oils. Carbohydrate content was similar in both products with lactose being the predominant source of carbohydrate. Micronutrient content was also similar between formulas evaluated in this analysis.

Weights and lengths were obtained by study staff at 2 weeks, 1, 2, 3, and 4 months of age using standardized procedures. Weight measurements were repeated until reproducible within 10 g on an electronic scale, and length measurements were repeated until reproducible within 0.5 cm. The average weight and length measurements were then recorded. Daily formula intake for the 2 days prior to study visits at 1, 2, 3, and 4 months was captured using daily records kept by caregivers and reviewed by clinical staff at study visits.

All studies included measurements of stool consistency and frequency at ages 1, 2, 3, and 4 months. For most studies, stool information was collected for each stool on daily records kept for 2 days prior to a study visit by parents/caregivers. For one study (in the CMF group), stool consistency was reported overall per day; thus, for analysis, the daily stool consistency was applied to each individual stool on that day. For all studies, stools were categorized by caregivers as being liquid, soft, formed, or hard.

Statistical analyses were performed using Stata (StataCorp. 2017. Stata Statistical Software: Release 15. College Station, TX: StataCorp LLC) and SAS/STAT software, version 9.4 (SAS Institute Inc., Cary, NC, USA). Primary analyses focused on the ITT population which included subjects who dropped out of the studies at any time. A separate PP analysis was also conducted including only infants who were compliant with the protocol throughout each study.

### 2.2. Anthropometrics

Summary statistics for infant weight, length, and weight-for-length percentile were evaluated by gender, study and treatment group. Models for weight and length change accounted for clustering within study. Multilevel models predicting weight, length, and weight-for-length percentile over time by gender included random effects for study and infant, and fixed effects for the baseline measure (baseline weight, length, or weight-for-height percentile), time, treatment, and the interaction of time and treatment. Weight-for-length percentiles were taken from WHO growth charts for ages 0 to < 2 years. Analyses were performed separately by ITT and PP status.

### 2.3. Stool Characteristics

Summary statistics for daily stool count and consistency per stool were conducted by study and by treatment group. Daily stool count was analyzed as a continuous outcome for each subject. Stool consistency was modeled as a multinomial outcome with four categories (liquid, soft, formed, and hard) over the course of the study visits (months 1, 2, 3, and 4). The longitudinal models for both stool frequency and stool consistency adjusted for group, time, and group by time interaction, and also included random effects for study and infant; post-hoc tests at each time point compared the marginal probability of each stool consistency between the two formula groups. Alpha was adjusted to 0.003125 to account for multiple comparisons for stool consistency (4 consistency categories at 4 different timepoints). Marginal probabilities were converted to percentages for presentation and discussion. Sensitivity analyses dropping the single PHF-W study with an added prebiotic [5] were conducted as this ingredient may affect stooling properties.

### 2.4. Intake

Longitudinal models for daily intake volume were used, including fixed effects for gender, baseline weight, time, group and group by time interaction, and random effects for study and infant. Post hoc tests were conducted at each month; alpha was adjusted to 0.0125 for comparisons at each of the 4 time points.

## 3. Results

Data from 511 infants were included (ITT: 197 CMF, 314 PHF-W). For the PP analysis, data were limited to the 400 infants (167 CMF, 233 PHF-W) who were compliant with the study protocol throughout each study. There was no significant difference in the number of infants who did not qualify for the PP analyses between the two groups (14% CMF, 20% PHF-W; p=0.367). One study of CMF did not collect breastfeeding history [6]. Of the remaining studies, the number of infants who received breastmilk before study enrollment was comparable (28% CMF, 32% PHF-W, and p=0.398). The rate of Cesarean section deliveries was 41% in infants fed CMF as compared to 30% in PHF-W (p=0.011).

### 3.1. Anthropometrics

There were no differences in weight gain (g/d) between PHF-W and CMF-fed infants. ITT boys fed PHF-W gained 28.8±5.8 g/day as compared to 27.7±5.4 in ITT CMF-fed boys (p=0.147); PP analysis also showed no significant differences between groups. For ITT girls, weight gain in the PHF-W group was 24.2±5.1 g/day compared to 23.8±4.9 in CMF-fed girls (p=0.542); PP analysis for the girls was also similar. There was no difference in girls' length gain (cm/month) for both ITT and PP analyses. ITT PHF-W-fed boys had greater length gain (+0.016 cm/month; p=0.017), and PP analysis also showed statistically significant greater length gain (+0.015 cm/month; p=0.043).

**Figure 1:**
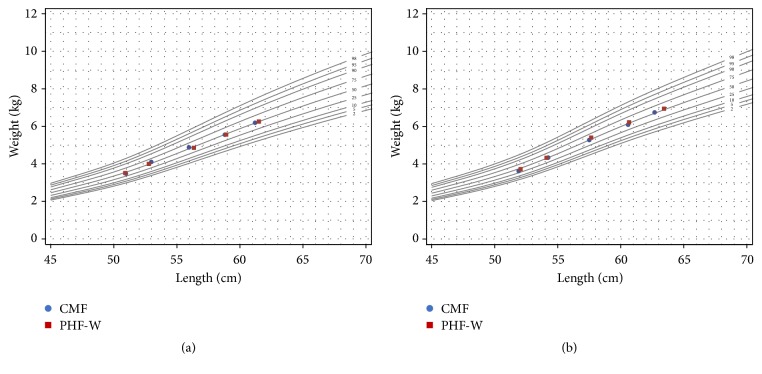
(a) Weight-for-length for girls. Mean weights and lengths at ages 2 weeks-4 months were plotted on WHO weight-for-length growth curves. Both formula groups demonstrate adequate growth that was clinically comparable. (b) Weight for-length for boys. Mean weights and lengths at ages 2 weeks-4 months were plotted on WHO weight-for-length growth curves. Both formula groups demonstrate adequate growth that was clinically comparable.

There were no differences between groups for boys or girls in WHO weight-for-length percentiles (Figures 1(a) and 1(b)). For boys, at baseline, the mean weight-for-length percentile was 38^th^ for ITT CMF-fed boys compared to 41^st^ for PHF-W; similar values were seen for PP analyses. At 1, 2, 3, and 4 months, the weight-for-length percentiles ranged from 50^th^ to 55^th^ for PHF-W fed ITT boys and 48^th^ to 52^nd^ for ITT CMF boys. For ITT girls, at baseline, both PHF-W and CMF groups were at the 43^rd^ percentile for weight-for-length. From 1 to 4 months of age, the weight-for-length percentiles ranged from 47^th^ to 50^th^ for PHF-W fed ITT girls and 49^th^ to 57^th^ for CMF-fed ITT girls.

### 3.2. Stool Characteristics

**Figure 2:**
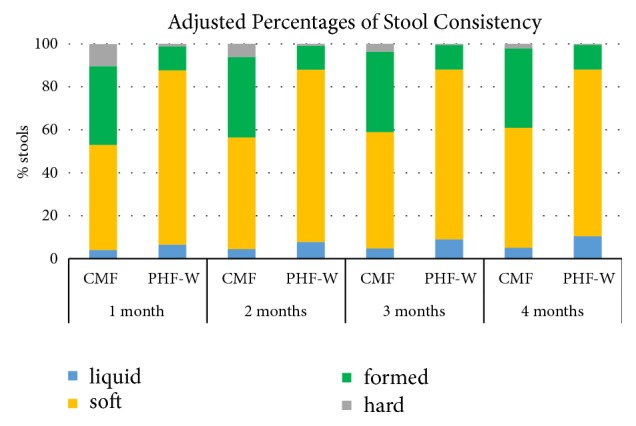
Graph represents adjusted percentages from individual participant data of 7 growth studies where infants were exclusively formula-fed PHF-W or CMF (Nan) from 2 weeks of age to 4 months. For 2 days before study visits at 1, 2, 3, and 4 months of age, caregivers kept a daily diary and recorded the consistency of each stool passed. Statistical significance was determined using marginal probabilities for stool consistency based on a longitudinal multinomial regression model with adjustment for treatment, age and treatment-by-age interaction, and accounting for clustering within study and infant. Statistical differences were observed for soft, formed, and hard stools at all time points.

Using a multinomial model treating the four stool consistency categories as the outcome, infants fed PHF-W had a significantly higher percentage of soft and lower percentage of formed and hard stools as compared to infants fed CMF at all time points (p<0.001; Table 2). Unadjusted percentages of soft stools for PHF-W were 79%, 80%, 78%, 76% at 1, 2, 3, and 4 months as compared to 49%, 44%, 48%, and 56% for CMF. For hard stools, the unadjusted percentages were 1% for months 1, 2, and 3 and <1% at 4 months for PHF-W (Figure 2). In the CMF group, the unadjusted percentages of hard stools were 11% at 1 month, 4% at 2 months, and 3% at 3 and 4 months (Table 2). PP results were similar. The sensitivity analysis showed that the prebiotic-containing-PHF-W study [6] did not affect stool consistency. Stool frequency was similar between groups at all time points.

### 3.3. Intake

For all infants combined, adjusting for gender and baseline weight, the increase in formula intake over time was greater for the PHF-W group than for the CMF group. However, in sex-stratified analyses, this difference was only evident among girls; there were no significant differences in the amount of formula intake by boys over time between the two formula groups (group by time interaction p=0.168). For girls the increase in volume intake over time was greater for PHF-W (group by time interaction p=0.006).

## 4. Discussion

This pooled analysis confirms that both PHF-W and CMF adequately support growth during early infancy and conclusively supports clinically relevant observations that infant formulas with a PHW matrix beneficially affects stool consistency. Growth was comparable between the two groups. Differences exist in stool consistency of infants fed PHF-W and CMF, with softer stools being reported significantly more often in PHF-W-fed infants and hard stools being reported significantly less often.

The two formulas studied here are isocaloric with composition described in Table 1. Milk-based infant formulas commercialized worldwide have protein content consistent with regulatory guidance, providing at least 1.8 g protein/100 kcal [12–15], with the majority of commercially available routine milk-based formulas having a caloric density of 20 kcal/oz and 1.8-2.2 g protein per 100 kcal. Both formulas used in this pooled analysis contained protein content within these ranges, with the PHF-W having a higher protein content (2.2 g/100 kcal) as compared to the CMF (1.8 g/100 kcal). Growth was comparable as weight gain (g/day) was within 3g/day between groups. While a statistical difference was seen in monthly length gain where boys fed PHF-W had greater gains than those fed CMF (+0.016 cm/month), the clinical relevance of this is uncertain. Weight-for-length percentiles were similar between the groups, indicating similar proportional growth between groups. Additionally, weight-for-length WHO percentiles tracked closely along the 50^th^  %ile throughout the study feeding period in both groups. Longer-term follow-up (to 10 years of age) has demonstrated that infants fed PHF-W with a protein level of 2.2 g/100 kcal have similar BMI trajectories to children who were exclusively breastfed in infancy [16].

Over the entire four month period combined, there was a significant difference between the prevalence of soft stools between infants fed CMF and PHF-W with the majority of the infants in the PHF-W group reporting soft stools at 1, 2, 3, and 4 months but less than half of CMF-fed infants reporting soft stools. This supports the results seen in a recent crossover study of formula-fed infants fed a similar PHF-W formula and a commercially available CMF in the United States with a higher proportion of casein than the CMF used in this pooled analysis [17]. In that crossover study with feeding periods of 2 weeks on each formula, significantly more soft stools were observed in the second week of feeding when subjects were fed PHF-W as compared to when they were consuming a CMF with a 60:40 whey:casein ratio. Other outcomes including vomiting and reflux were not collected in a uniform manner in these trials. Including more outcomes related to tolerance in future studies will provide a more complete picture of caregiver-perceived formula tolerance.

Hard stools are rarely observed in exclusively breastfed infants, and firm or hard stools are often seen with the change from breast milk to standard infant formula or after the introduction of solids [18]. Constipation is more frequent in casein-predominant than in whey-predominant formulas, and hydrolyzed formulas produce more frequent and softer stools [18]. Results of this pooled analysis provide further evidence that partially hydrolyzed formulas can induce softer stools and fewer hard and formed stools than standard infant formula in nonconstipated infants. In this pooled analysis, no difference in stool frequency was observed while stools were reported to be softer in the partially hydrolyzed whey protein-based formula compared to a formula with a protein base consisting predominantly of intact whey. Whether consuming an intact casein-predominant infant formula may result in stool frequency and consistency different than that of an intact whey-dominant infant formula like the one studied here is unknown.

Beyond protein, fat blends have been demonstrated to affect stool consistency [19, 20]. In a study of formulas from another manufacturer, softer stools were also observed in infants fed a partially hydrolyzed whey formula compared to a standard intact cow's milk formula, though the partially hydrolyzed whey group also contained a prebiotic and an alternate fat source than the standard formula studied [21]. However, in the formulas used in this pooled analysis, the fat blends were similar so that differences observed are most likely attributed to the difference in the protein matrix and not fatty acid composition and configuration.

Greater formula intake over time was also observed in the PHF-W-fed girls. Subjects in the PHF-W group consumed an average of 25 ounces/day at 1 month up to an average of 32 ounces/day at 4 months of age which is comparable to another study of partially hydrolyzed whey-based formula in the US in which infants consumed 24 ounces/day at 28 days of age up to 29.5 ounces/day at 84 days [22]. Wu and colleagues examined infants who were randomized to receive an intact whey:casein formula (61:39) or a partially hydrolyzed whey:intact casein (63:37) formula, and similarly saw greater formula intake in the group receiving partially hydrolyzed whey though no differences were observed in growth or stool consistency [23].

Statistical differences in formula intake in girls highlight one limitation of this analysis which is that all of the PHF-W studies were conducted in the US while the CMF studies were conducted in Italy and France. The impact of cultural differences and feeding patterns in the US that may have affected formula intake is not known. Parenting styles among various countries could differ in terms of volume of formula offered, bottle size, responsiveness to satiety cues of infants, and prompting infants to finish entire bottles. In addition, the rate of Ceserean-section deliveries was significantly higher in the CMF-fed group, which again could be due to differences in medical practices among different countries.

The strength of this pooled analysis lies in the similarity of the study design across all seven pooled studies. Data collection timepoints were identical as were the categories of stool consistency. Anthropometric measurements were also taken in a standardized fashion among all of the studies. The protein matrix within all of the PHF-W studies and within all of the CMF studies were identical, and formulas were isocaloric. However, the CMF used in the pooled analysis offers a more conservative comparison than to intact formulas available in the US as the protein content of the major intact CMF in the US have a higher overall protein and casein content than that of the formula used in this pooled analysis.

## 5. Conclusions

In conclusion, infants fed PHF-W grow similarly to those fed CMF with both formulas supporting adequate growth in infancy. This information is reassuring to healthcare professionals when discussing formula choices with their patients. Differences in stool consistency were observed between infants fed PHF-W versus CMF with PHF-W fed infants having a higher proportion of soft stools and lower proportion of formed and hard stools than those fed CMF. This difference may be meaningful to caregivers and useful for healthcare professionals to better inform their discussions when exclusive breastfeeding is not possible.

## Figures and Tables

**Table 1:** Nutrient composition of infant formulas.

**Composition**	**Units**	**CMF**	**PHF-W**
Energy	kcal/100 mL	67	67
Carbohydrate	g/100 kcal	11	11.2
Fat	g/100 kcal	5.3	5.1
Protein	g/100 kcal	1.8	2.2
Whey:Casein	-* *-* *-	70:30	100:0

Formulas tested in different trials had consistent energy and macronutrient composition but may have varied slightly on micronutrients. The exception is one PHF-W study which had a protein content of 2.4 g/100 kcal [9]. None of the formulas used included probiotics. One study of PHF-W included 4 g galactooligosaccharides/L formula [5]; no other study formulas contained a prebiotic.

**Table 2:** Adjusted percentages (95% CIs) of stool consistency at 1, 2, 3, and 4 months, by formula group based on a pooled analysis of individual data from four Nestlé PHF-W (Good Start) growth studies and 3 Nestlé CMF (Nan) growth studies.

**Age**	**Consistency Category**	**Actual Pooled Percentage of Stool Consistency (**%**)**		**Adjusted Percentage of Stool Consistency and 95% CI (**%**)**		**p-value**
		**CMF**	**PHF-W**	**CMF**	**PHF-W**	
1 month	Liquid	4%	7%	4.1% (2.2, 6.1)	6.6% (4.5, 8.8)	0.073
	Soft	49%	79%	48.9% (45.6, 52.2)	81.1% (78.6, 83.5)	**<0.001**
	Formed	31%	11%	36.7% (33.6, 39.9)	11.1% (9.5, 12.6)	**<0.001**
	Hard	11%	1%	10.2% (8.1, 12.3)	1.2% (0.6, 1.7)	**<0.001**

2 months	Liquid	4%	5%	4.5% (2.6, 6.4)	7.8% (5.5, 10.0)	0.022
	Soft	44%	80%	52.0% (49.6, 54.4)	80.2% (77.9, 82.4)	**<0.001**
	Formed	42%	11%	37.4% (35.2, 39.6)	11.2% (10.2, 12.3)	**<0.001**
	Hard	4%	1%	6.1% (5.1, 7.2)	0.8% (0.5, 1.1)	**<0.001**

3 months	Liquid	5%	8%	4.8% (6.5, 11.6)	9.1% (6.5, 11.6)	0.007
	Soft	48%	78%	54.2% (51.6, 56.8)	79.0% (76.5, 81.5)	**<0.001**
	Formed	36%	11%	37.4% (34.9, 39.8)	11.4% (10.2, 12.6)	**<0.001**
	Hard	3%	1%	3.6% (2.6, 4.6)	0.5% (0.2, 0.8)	**<0.001**

4 months	Liquid	4%	10%	5.1% (2.7, 7.5)	10.5% (7.5, 13.6)	0.005
	Soft	56%	76%	55.9% (52.0, 59.7)	77.6% (74.4, 80.8)	**<0.001**
	Formed	31%	12%	36.9% (33.3, 40.6)	11.5% (9.6, 13.3)	**<0.001**
	Hard	3%	<1%	2.1% (1.2, 3.0)	0.4% (0.1, 0.7)	**<0.001**

Adjusted percentages are rescaled marginal probabilities of stool consistency obtained from a longitudinal multinomial regression model with adjustment for treatment, age and treatment-by-age interaction, and accounting for clustering within study and infant. P-values are based on comparisons of the marginal probability of stool consistency at each time point. Based on the number of multiple comparisons made, a p-value of <0.003 is considered statistically significant. Actual (unadjusted) pooled percentages are also presented here; some stools were reported but missing consistency categorization.

## Data Availability

The datasets generated and/or analysed during the current study are not publicly available due to the proprietary nature of the data but may be made available from the corresponding author on reasonable request. Datasets are housed within files kept by Nestlé Nutrition.
